# Structural and functional properties of bacterial communities associated with rootless duckweed (*Wolffia globosa*) and their effect on the *Wolffia* growth

**DOI:** 10.1186/s40793-025-00759-6

**Published:** 2025-08-07

**Authors:** Yuparat Saimee, Kousuke Kuwai, Hidehiro Ishizawa, Daisuke Inoue, Arinthip Thamchaipenet, Michihiko Ike

**Affiliations:** 1 Division of Sustainable Energy and Environmental Engineering, The University of Osaka, 2-1 Yamadaoka, Suita, Osaka 565-0871 Japan; 2 Department of Applied Chemistry, University of Hyogo, 2167 Shosha, Himeji, Hyogo 671-2280 Japan; 3 Department of Genetics, Faculty of Science, Kasetsart University, Bangkok, 10900 Thailand; 4 Duckweed Holobiont Resource & Research Center (DHbRC), Kasetsart University, Bangkok, 10900 Thailand

**Keywords:** *Wolffia* growth, *Wolffia* microbiome, Core taxa, Keystone taxa, Functional characteristics

## Abstract

**Background:**

Rootless duckweed, *Wolffia globosa*, is emerging as a high-protein and starch biomass resource for various applications. However, the microbiomes and functional properties associated with *Wolffia* across a wide range of microbial sources remain largely unexamined. This study investigates the structure and functioning of the *Wolffia* microbiome and its impact on plant growth.

**Results:**

A co-cultivation experiment with axenic *W. globosa* and bacterial communities derived from various sources revealed varied effects, that municipal wastewater-derived bacterial communities had a more pronounced positive effect on growth of *W. globosa* compared to those from pond water. 16S rRNA amplicon sequencing found that *Beijerinckiaceae*, *Caulobacteraceae*,* Comamonadaceae*,* Methylophilaceae*,* Rhizobiaceae*, and *Sphingomonadaceae* were consistently conserved and identified as core taxa in the *Wolffia* microbiome. Functional profiling indicated that genes related to bacterial colonization and adaptation to the rootless morphology contribute to selective microbiome recruitment, with enriched functions in motility, chemotaxis, flagella assembly, quorum sensing, and ABC transporters. In addition, it was found that *Bdellovibrionaceae*, *Beijerinckiaceae*, and *Sphingomonadaceae* may act as “hub microorganisms” and “keystone taxa,” shaping community structure and directly or indirectly influencing *Wolffia* growth.

**Conclusion:**

Collectively, the results of this study unveiled the robust core taxa and functional profiles of the *Wolffia* microbiome across diverse microbial sources, with certain taxa differing from those in rooted duckweed. This study comprehensively characterizes the *Wolffia* microbiome and enhances understanding of it, providing insights for developing efficient biomass production systems.

**Supplementary Information:**

The online version contains supplementary material available at 10.1186/s40793-025-00759-6.

## Background

Members of the family *Lemnaceae*, commonly known as duckweed, are floating monocots prevalent in slow-flowing freshwater ecosystems worldwide, from tropical to temperate regions [1, 2]. This family includes five genera divided into two subfamilies: Lemnoideae, which has roots (including *Landoltia*, *Lemna*, and *Spirodela*), and Wolffioideae, which lacks roots (including *Wolffia* and *Wolffiella*). Duckweeds are recognized for their high biomass production and valuable contents, thus promising for various applications, including human food, animal feed, bioenergy, and phytoremediation, providing a sustainable approach for water purification and biomass production [3–7].

*Wolffia* is the smallest rootless duckweed, with a globose or oval shape and a flattened upper surface [8–10]. It rapidly reproduces vegetatively (growth rate of 0.155 to 0.559 d^–1^) and has the shortest doubling time among higher plants (approximately 1–2 d) [11]. *Wolffia* biomass is rich in protein (20 to 30% of its dehydrated mass) and contains all essential amino acids in adequate quantities for adults. The total starch content of biomass ranges from 10 to 15% of dehydrated mass, and the fiber content is around 25%. The fat (1–5%) is mainly polyunsaturated fatty acids, which are beneficial for health and help address dietary imbalances [4, 12–14]. Unique to *Wolffia* is its vitamin B12 content and lack of calcium oxalate crystals, which cause digestive issues [15–17], making it a promising choice for human food and animal feed in addition to bioenergy production.

The microbial community associated with plants, known as the plant microbiome, establishes a beneficial relationship and functions collectively as a holobiont, enhancing the productivity or fitness of plants. Studies on rooted duckweeds have proven that their growth is significantly influenced by its microbiome [18–22]. Duckweed selectively recruits bacterial communities from its surrounding environment, shaping a unique microbiome with core conserved taxa, including *Caulobacteraceae*,* Comamonadaceae*,* Methylophilaceae*,* Oxalobacteraceae*, *Pseudomonadaceae*,* Rhizobiaceae*,* Rhodospirillaceae*, and *Sphingomonadaceae* [18–21]. Notably, *Comamonadaceae* and *Oxalobacteraceae* are commonly predominant taxa in duckweed, highlighting their significant roles in plant-microbe interactions [18, 19]. In addition, several duckweed-associated bacteria, more specifically plant growth-promoting bacteria (PGPB) isolated from rooted duckweed microbiomes, can enhance duckweed productivity and phytoremediation efficiency [23–27]. However, their beneficial effects can be inhibited by the co-presence of plant growth-inhibiting bacteria (PGIB) and complicated interactions within the microbiome [22, 24, 28, 29]. Thus, managing the microbiome is essential for sustainably improving duckweed productivity.

Despite extensive efforts on the microbiome of rooted duckweeds, knowledge on the microbiomes of rootless duckweeds is very limited and not systematically investigated [19, 20, 30]. Because rootless duckweeds have different morphology, their microbiome may function differently than those of rooted duckweeds [19, 20]. As rootless duckweeds can be a good source of biomass, it is important to understand the characteristics of *Wolffia* microbiome, including those of core taxa and functional properties of overall microbiome, that affect the growth of host plant.

This study has three main goals: (i) to assess how different bacterial communities affect the growth of *Wolffia*; (ii) to clarify the structure and functions of the bacteria associated with *Wolffia* and its core taxa; and (iii) to determine what factors cause changes in the *Wolffia* microbiome. Axenic *Wolffia globosa* was co-cultivated with 18 different bacterial communities derived from freshwater ponds, municipal secondary effluents, and activated sludges. Bacterial communities were analyzed using a 16S rRNA amplicon sequencing and diverse methods were applied to analyze the structure and function of these communities. This study aimed to shed light on the mostly unknown microbiome of rootless duckweeds and improve systems for producing its biomass efficiently.

## Materials and methods

### Recovery of bacterial communities from various sources

As the microbial source, surface water samples were collected from 10 freshwater ponds in September 2023, along with secondary effluent and activated sludge samples from 4 municipal wastewater treatment plants in Osaka, Japan, in December 2023 (Table S1). The activated sludge samples were pretreated to disperse the flocs as follows: the samples were left at room temperature to allow the sludge flocs to settle. After settling, 2.5 g-wet of the settled sludge was added to 250 mL of phosphate-buffered saline (PBS), shaken at 180 rpm and 28 °C overnight. To recover the bacterial communities from pond water, secondary effluent, and pretreated activated sludge, all samples were passed through a 3.0 μm membrane filter (MF-Millipore™, Merk Millipore, Darmstadt, Germany) to remove coarse particles, including fungi, microalgae, and larger activated sludge flocs. Sixty milliliters of the filtrate were then centrifuged at 10,000×*g* for 10 min at 4 °C to recover the bacterial communities. The bacterial communities were washed twice with a sterile modified Hoagland (MH) medium [31] and then re-suspended in the original volume of sterile MH medium for the co-cultivation experiment. The bacterial communities recovered from pond water, secondary effluent, and activated sludge were hereinafter referred to as categories PW, SE, and AS, respectively.

### Co-cultivation of *Wolffia* with bacterial communities

Axenic *W. globosa* (clone no. RDSC8152) was used as the test plant. The plant was sterilized with sodium hypochlorite, and the sterilized plant was maintained as previously described [32]. We have verified the absence of microorganisms routinely and prior to use in the co-cultivation experiments by confirming no microbial growth occurred on R2A agar “DAIGO” (Shiotani M.S. Co., Ltd., Hyogo, Japan) after applying sterile *W. globosa* with and without homogenization to the plates. The plants were cultured in MH medium for 5 d prior to the experiment. Twenty fronds of the precultivated *Wolffia*, which included 10 fronds from attached mother-daughter pairs and 10 single fronds, were transferred into a flat-bottomed 6-well plate containing 10 mL of bacterial community suspensions. The inoculated fronds occupied an area of approximately 0.29–0.30% of the well surface. The plates were then cultured for 10 d in a growth chamber set to 28 °C, with a light intensity of 80 µmol/m^2^/s, and a photoperiod of 16 h light/8 h dark. The cultivation period was divided into two stages: (i) the colonization stage and (ii) the growing stage. The colonization stage lasted for the first 5 d to allow the bacterial community to attach to and colonize the *Wolffia*. Evidence suggests that 5 d is sufficient for the duckweed microbiome to stabilize [18, 19, 33]. Subsequently, all *Wolffia* were transferred to 10 mL of fresh bacteria-free MH medium to continue the growing stage. During this stage, the MH medium was renewed on day 8 to address nutrient deficiency. After 10 d of cultivation, the *Wolffia* were collected, excess liquid on the fronds was blotted with sterile tissue paper, and the fronds were placed in sterile microtubes for further experiments. The medium was also collected for water quality measurements. Axenic *W. globosa* cultivated in MH medium without any bacterial communities served as the experimental control. We performed five repetitions for each sample and the control.

### Evaluation of *Wolffia* growth

To measure the growth of *Wolffia* periodically, the frond area was recorded at 0, 3, 5, 7, and 10 d. We ensured consistency by photographing using the same equipment and maintaining the same distance, lighting, and time. The photos were analyzed using Collaboratory Software (https://colab.research.google.com; accessed in September 2023) to detect the green parts of the plant and calculate the relative growth rate (RGR). In brief, the analysis process involved converting the images from BGR to HSV color space. A mask was applied to isolate the green areas of the plant, and we counted the number of white pixels in the masked image that represented these green areas. This count was then used to determine the percentage of green pixels compared to the entire surface of the image, which reflects the frond area of *Wolffia*. The percentage of the green area was subsequently used to calculate *Wolffia* RGR. The increase or decrease in plant growth was assessed using the effect on plant growth (EPG) value, which is derived from the RGR compared to the control, and the EPG values greater than + 5%, between ‒5% and + 5%, and less than ‒5% were judged as promoting, neutral, and inhibitory effects, respectively, as previously described [22]. The method for calculating EPG is provided in the supplementary information (Text S1).

### Determination of physicochemical water quality parameters

Water temperature and pH were measured at the sampling points using a portable pH meter (TPX–999i; Toko Kagaku, Tokyo, Japan). Dissolved oxygen (DO) was measured with a portable galvanic DO sensor (DO–30; Kasahara Chemical Instruments, Saitama, Japan). Additionally, dissolved organic carbon (DOC), total dissolved nitrogen (TDN), and phosphorus as phosphate (PO_4_–P) in the collected water and the medium after co-cultivation were analyzed as environmental parameters. Samples were filtered through a 0.45 μm cellulose acetate filter (Advantec, Tokyo, Japan) before analysis. DOC and TDN were measured using a total organic carbon analyzer (TOC–L, Shimadzu, Kyoto, Japan) equipped with a total nitrogen measuring unit (TMN–L, Shimadzu). PO_4_–P was analyzed using ion chromatography (IC) with a Shimadzu SLC-10Avp system (Shimadzu), which included a CDD-10Asp detector (Shimadzu) and an IonPac AS4A-SC column (4 × 250 mm, Thermo Scientific, Massachusetts, USA). The eluent was composed of 1.8 mM Na_2_CO_3_ and 1.7 mM NaHCO_3_, delivered at a flow rate of 1.0 mL/min.

### DNA extraction and 16S rRNA amplicon sequencing

Indigenous bacterial communities from the original microbial sources were filtered through a 0.2 μm membrane filter (Toyo Roshi Kaisha, Tokyo, Japan). The filtrates were subjected to DNA extraction. After 10 d of co-cultivation, *Wolffia* samples were homogenized using a BioMasher II (Nippi, Tokyo, Japan). Microbial DNA from all samples was extracted using the Cica Geneus DNA Extraction ST kit (Kanto Chemical, Tokyo, Japan). The extracted DNA was subjected to 16S rRNA amplicon sequencings targeting the V5-V6 region of the bacterial 16S rRNA gene with primers 799 F [34] and 1185mR [35]. Sequencing was performed on an Illumina MiSeq sequencer (Illumina, CA, USA) at the Bioengineering Lab. Co., Ltd. (Kanagawa, Japan).

### Data processing and analysis

The raw reads from 36 samples were processed using the QIIME2 version 2023.7 pipeline, which included 18 original microbial source samples designated S_PW_ (with letters A-J for category PW) and S_SE_ and S_AS_ (with numbers 1–4 for categories SE and AS). Eighteen *Wolffia* microbiome samples were designated as WgPW_, WgSE_, and WgAS_, using the same letter and number designations as their corresponding original microbial source samples. The obtained sequences were processed using QIIME 2.0; the raw reads were demultiplexed and filtered, high-quality reads were merged, and chimeric sequences were removed. The complied high-quality sequences were clustered into amplicon sequencing variants (ASVs) using the DADA2 plugin [36]. Then, ASVs were assigned to a taxonomy by the QIIME2 feature-classifier plugin based on the SILVA SSU Ref NR database v138. ASVs whose sequences were not present in the database and cyanobacterial ASVs were excluded. Finally, an abundance table combined with taxonomy information was generated. Rarefaction analysis was performed based on the feature table, with random sampling to the minimum sequencing depth (19,180) of all samples using the q2-diversity plugin. The Shannon and Chao1 indices were used as an index of alpha diversity, and one-way analysis of variance (ANOVA) with Honestly Significant Difference (Tukey HSD) test was conducted for statistical differences. Beta diversity of bacterial communities was analyzed using principal-coordinate analysis (PCoA) based on the Bray-Curtis matrix in R version 4.3.2 with the “vegan” package to visualize the compositional dissimilarity in community structure between groups of samples. Permutation multivariate analysis of variance (PERMANOVA) [37] was conducted to assess the statistical significance of the observed differences in microbiome structure between groups. Data were visualized using the “ggplot2,” “VennDiagram,” and “ComplexHeatmap” packages in R.

To explore the environmental factors or bacterial families that drive the structural variation in *Wolffia* microbiomes and the original microbial sources, redundancy analysis (RDA) and partial RDA were conducted using the “vegan” package in R [38]. Forward selection was performed with the “forward.sel” function from the “packfor” package to identify the most significant explanatory variables in relation to the microbiome structure [39]. Details of parameter setting and statistical significance evaluation are provided in supplementary information Text S2.

Functional profiling of the original microbial sources and *Wolffia* microbiomes was predicted based on ASVs using the PICRUSt2 algorithm [40] through QIIME2. The predicted functional genes were categorized into pathways based on the Kyoto Encyclopedia of Genes and Genomes (KEGG) orthologs [41]. For gene categories, functions with a mean relative abundance > 0.4% were categorized into tier 3, normalized among the 36 communities, and the z-scores were plotted using the “ComplexHeatmap” package in R.

To identify the core microbiome of *Wolffia*, the ASVs with an occurrence frequency greater than 0.7 [42] in each *Wolffia* category (i.e., WgPW, WgSE, and WgAS), regardless of their relative abundance, were defined as core *Wolffia* taxa. Data were transformed using centered log ratio (clr) transformation, where the abundance counts were compared to their geometric mean. Taxa with clr values close to 0 indicated their average abundance. The transformed data were displayed using the “ComplexHeatmap” package in R.

The potential key players influencing *Wolffia* growth were identified based on its relationship with the relative abundance of bacterial families. Pearson correlation was constructed using the “metan” package in R and visualized using the “corrplot” package based on hierarchical clustering. Positive and negative correlations with Wolffia growth were then visualized as scatter plots.

## Results

### Effect of bacterial communities on *Wolffia* growth

**Fig. 1:**
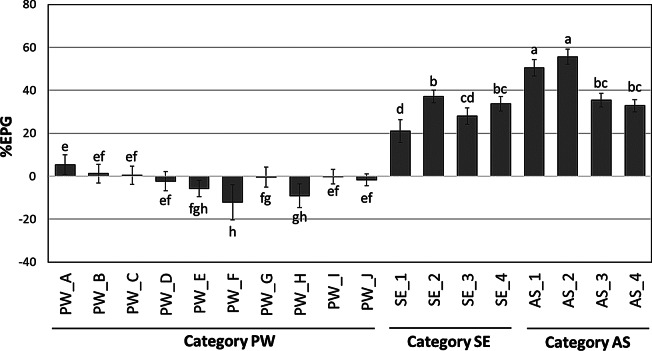
Effects on plant growth (EPG) of bacterial communities from categories PW, SE, and AS for 10 d cultivation term. Error bars represent standard deviations (*n* = 5). Bars marked with different letters indicate significant differences (*p <* 0.05, Tukey’s HSD test)

The RGR of axenic *Wolffia* without any bacterial communities (the experimental control) was 0.328 ± 0.015 d^− 1^ under the experimental settings of this study. Bacterial communities recovered from three categories of microbial sources (PW, SE, and AS) had different effects on *Wolffia* growth, namely inhibitory, neutral, or promoting effects (Fig. 1). Six of ten communities in the category PW had neutral effects on *Wolffia* growth, while three (PW_E, PW_F, and PW_H) reduced growth by up to ‒12.2% without visible disease symptoms or chlorosis (Fig. S1). Only community PW_A in this category showed a slight promoting effect (+ 5.3%), marginally exceeding the threshold for promotion (+ 5%). In contrast, communities from AS and SE categories significantly promoted growth, with the EPG ranging from + 21% to + 55.6%, resulting in *Wolffia* growth of up to 1.6-fold than those from PW.

This study used a two-stage cultivation system, allowing bacterial colonization before transferring *Wolffia* to a fresh axenic medium, with medium renewal on day 8 to prevent nutrient depletion that could lead to turion production. Monitoring *Wolffia* growth from day 5 to day 10 revealed a shift in SE communities that transitioned from inhibitory to promoting effects, while AS communities consistently maintained the growth-promoting effects (Fig. S2).

### Diversity and taxonomic composition of *Wolffia* microbiome in comparison with bacterial communities in microbial sources

Through 16S rRNA amplicon sequencing of 18 microbial sources and 18 *Wolffia* microbiota, 1,071,728 reads were obtained, identifying 5,699 ASVs (Table S2, S3). Among the microbial sources, SE had the highest number of ASVs (2,847), followed by PW (1,504) and AS (1,102). The *Wolffia* microbiome contained 1,077 ASVs, with only 1.7–5.5% overlapping with those from their microbial sources (Fig. S3).

**Fig. 2:**
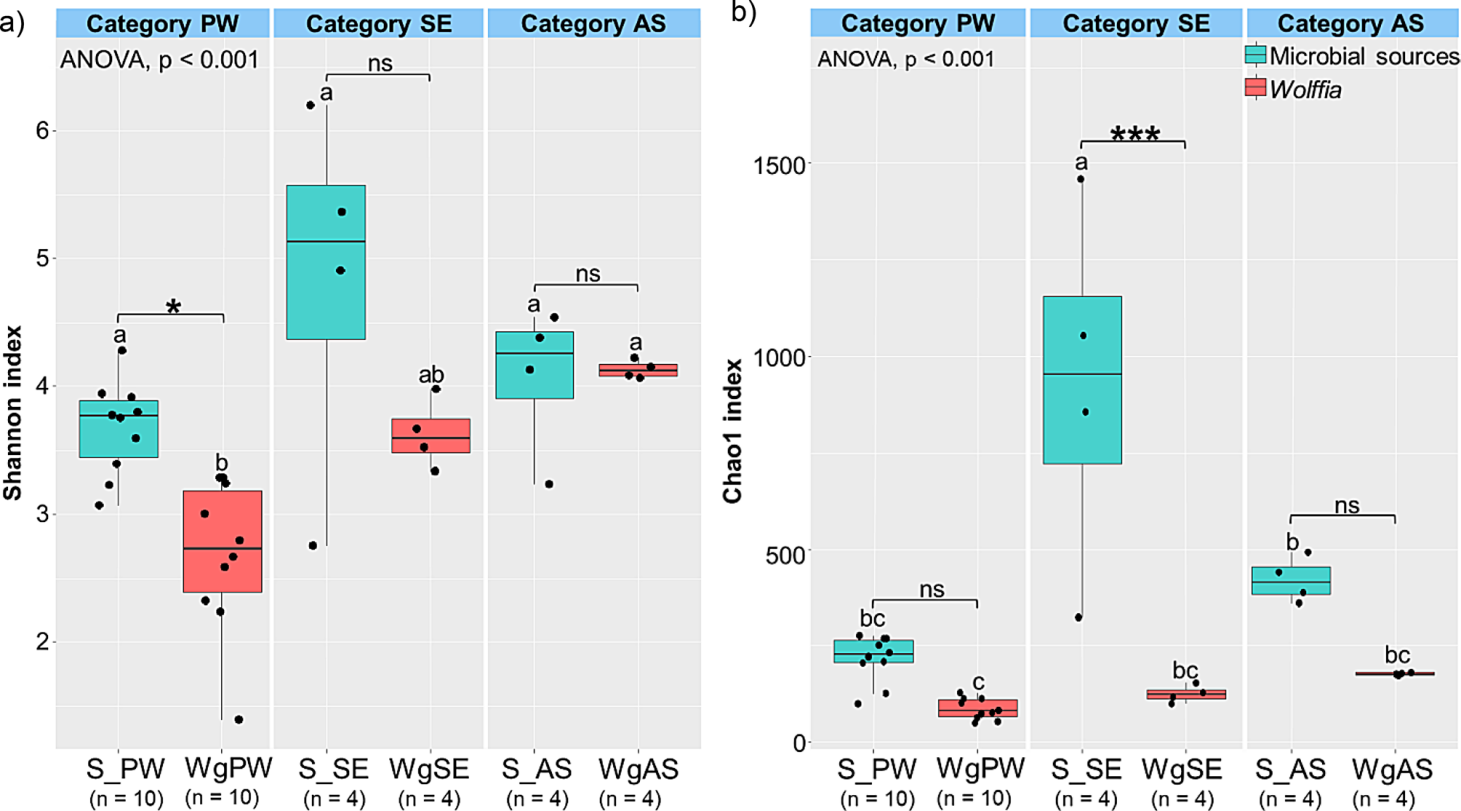
Alpha diversity based on **a**) Shannon and **b**) Chao1 indices. Letters indicate significant differences among groups within and across categories. Asterisks (*) denote significant differences between *Wolffia* and microbial sources within the same category (ANOVA Tukey’s HSD test, *p < 0.05, ***p < 0.001, *ns*: no significant difference)

Alpha diversity analysis using the Shannon and Chao1 indices revealed significant differences between sample groups (ANOVA, *p* < 0.001) (Fig. 2). *Wolffia* groups commonly displayed lower diversity and richness compared to their microbial sources, with significant differences (Tukey HSD test, *p* < 0.05) noted only between WgPW and S_PW for the Shannon index (Fig. 2a) and between WgSE and S_SE for the Chao1 index (Fig. 2b). These findings suggest that *Wolffia* selectively assembles specific bacterial populations from its microbial sources, resulting in reduced bacterial diversity.

PCoA based on the Bray-Curtis matrix confirmed that *Wolffia* microbiota were congregated with small separations and formed distinct clusters from microbial sources (Fig. S4). PERMANOVA further demonstrated that the *Wolffia* microbiome significantly differed from their microbial sources (R^2^ = 0.14145, *p* = 0.001), and its compositional structure varied across the microbial sources (R^2^ = 0.29035, *p* = 0.001), suggesting that *Wolffia* selectively harbors specific taxa depending on the microbial source.

**Fig. 3:**
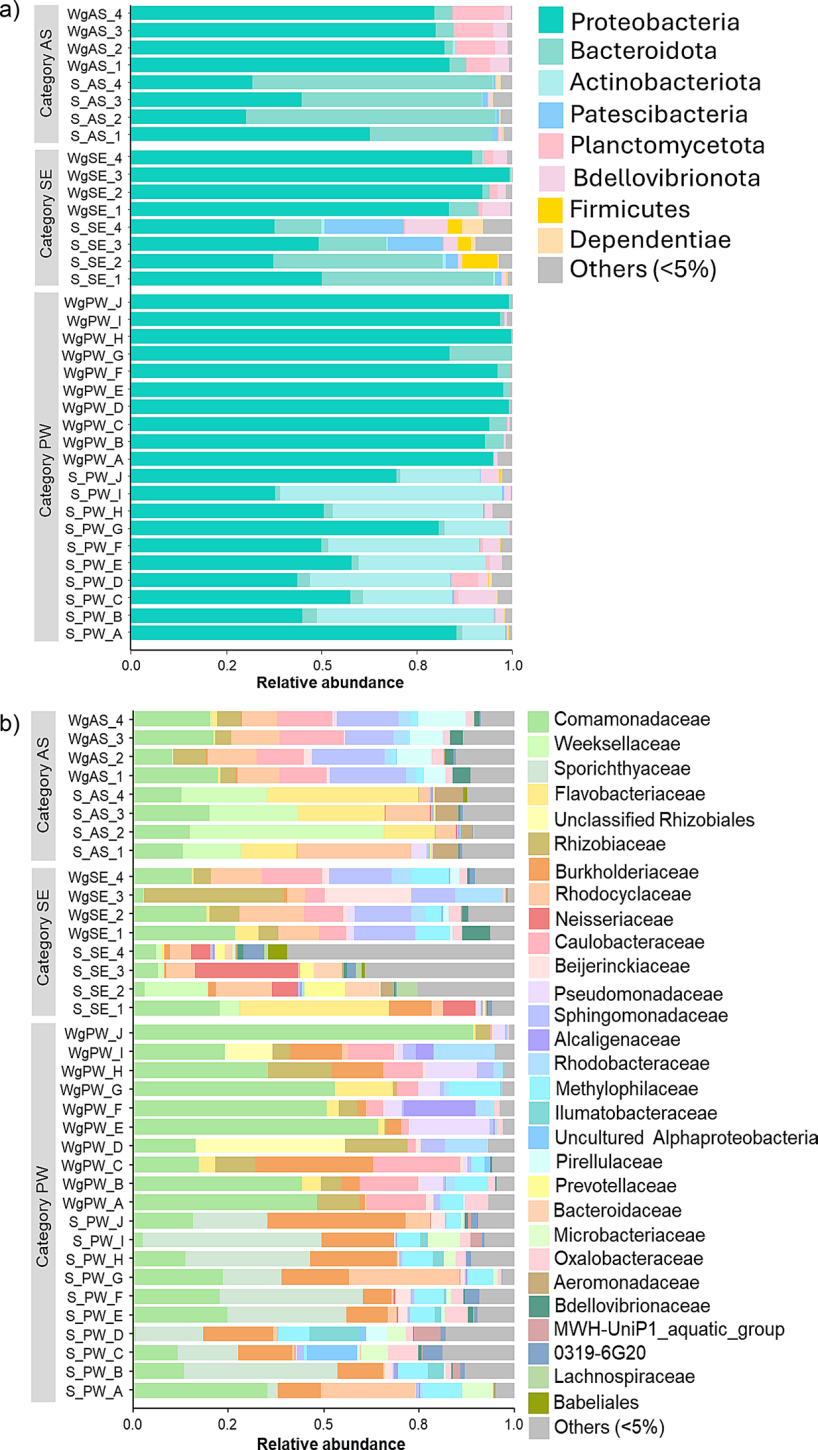
Taxonomic composition of bacterial communities in microbial sources and *Wolffia* microbiome at **a**) phylum and **b**) family levels. Others include all the classified and unclassified taxa with relative abundance of less than 5% in the sample

The taxonomic distribution of bacterial communities highlighted notable differences between *Wolffia* microbiomes and their microbial sources across all three categories. *Wolffia* displayed reduced phyla diversity but increased abundance of specific phyla compared to the corresponding microbial sources (Fig. 3a). *Proteobacteria* was the most dominant phylum in microbial sources, comprising 37.9–85.3% in S_PW, 37.4–50.0% in S_SE, and 30.2–62.5% in S_AS. In contrast, *Wolffia* microbiomes had a significantly higher abundance, ranging from 83.6 to 99.9% in WgPW, 83.4–99.2% in WgSE, and 79.6–83.6% in WgAS. Other prevalent phyla in microbial sources included *Actinobacteriota* in S_PW (11.4–58.1%) and *Bacteroidota* in S_SE (12.2–44.9%) and S_AS (32.2–62.5%). Notably, *Actinobacteriota* was absent in *Wolffia* microbiota, while *Planctomycetota* was uniquely dominant in WgAS (6.4–13.6%). At the class level, microbial sources mainly consisted of *Gammaproteobacteria* (23.4–82.9%) and *Actinobacteria* in S_PW (11.3–56.0%) in S_PW, and *Bacteroidia* in S_SE (12.1–44.9%) and S_AS (32.0–65.3%) (Fig. S5a). In *Wolffia* microbiomes, *Gammaproteobacteria* (9.6–92.4%) and *Alphaproteobacteria* (6.1–89.6%) were dominant across all categories, except in WgAS, where *Planctomycetes* was also prominent at 6.2–13.4%.

At the order and family levels, differences in dominant taxa between *Wolffia* and its microbial sources became more apparent (Fig. S5b and Fig. 3b). At the family level, distinct community compositions between *Wolffia* and its microbial sources were observed, with the dominant families varying by category. In S_PW, *Sporichthyaceae* (2.8–47.1%), *Burkholderiaceae* (7.8–36.1%), and *Comamonadaceae* (0.8–35.2%) were the three predominant families, followed by *Rhodocyclaceae* (< 0.1–28.9%), *Ilumatobacteraceae* (< 0.1–13.3%), and *Methylophilaceae* (0.9–10.4%). In WgPW, while *Comamonadaceae* was the most dominant family (16.5–89.0%) and *Burkholderiaceae* maintained similar abundance levels (< 0.1–30.7%), the other predominant families in S_PW showed a significant reduction. Instead, *Caulobacteraceae* (0.4–22.8%), *Pseudomonadaceae* (< 0.1–21.0%), *Rhizobiacea*e (0.4–16.5%), *Rhodobacteraceae* (< 0.1–15.7%) and unclassified *Rhizobiales* (< 0.1–39.1%) were predominated in WgPW. In particular, *Pseudomonadaceae* and *Rhizobiaceae* undetectable in S_PW, were exclusively enriched in significant abundance in WgPW. In category SE, *Neisseriaceae* (4.9–27.2%), *Comamonadaceae* (3.3–22.7%), *Flavobacteriaceae* (0.2–39.1%), *Weeksellaceae* (1.6–16.4%), and *Rhodocyclaceae* (2.9–14.6%) predominated in the microbial sources (S_SE). In contrast, WgSE was dominated by *Rhizobiaceae* (4.4–36.7%), *Comamonadaceae* (2.6–26.7%), *Rhodocyclaceae* (4.8–16.8%), *Sphingomonadaceae* (11.2–16.5%), and *Caulobacteraceae* (5.2–16.0%). Also, *Beijerinckiaceae* (0.2–22.7%), *Rhodobacteraceae* (0.5–12.2%) and *Methylophilaceae* (0.2–9.6%) were commonly present. Most of these families, other than *Comamonadaceae* and *Rhodocyclaceae*, were significantly enriched on *Wolffia*. In category AS, the microbial source (S_AS) was dominated by *Weeksellaceae* (15.3–51.0%), *Flavobacteriaceae* (13.2–39.8%), and *Rhodocyclaceae* (2.6–29.8%), followed by *Comamonadaceae* (12.7–20.0%) and *Aeromonadaceae* (2.9–7.4%). In contrast, *Wolffia* (WgAS) exhibited distinct composition, with *Comamonadaceae* (10.4–22.3%), *Sphingomonadaceae* (12.7–20.0%), and *Caulobacteraceae* (12.4–16.8%) as the three most dominant families. *Pirellulaceae* (5.6–12.7%), *Rhodocyclaceae* (9.0–12.7%), and *Rhizobiaceae* (4.1–8.6%) were also prevalent in WgAS.

### Core taxa in *Wolffia* microbiome

**Fig. 4:**
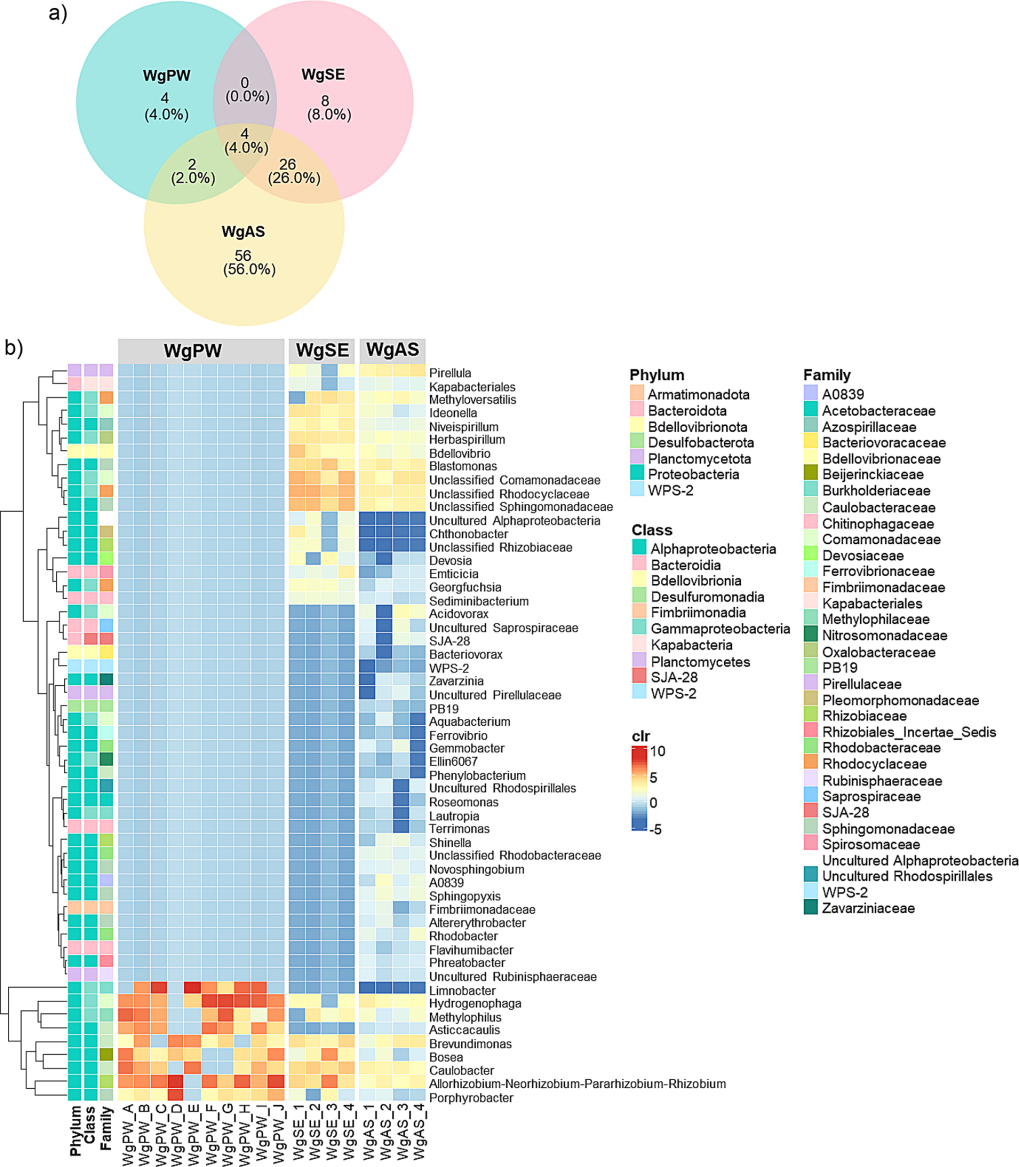
Core taxa of the *Wolffia* microbiome defined by ASVs with an occurrence frequency greater than 0.7 per *Wolffia* category, regardless of their relative abundance. **a**) Venn diagram showing shared and unique ASVs and **b**) heatmap presenting the 55 core genera

The core microbiome of *Wolffia* was identified by selecting taxa with an occurrence frequency greater than 0.7 in each category, irrespective of their relative abundance. This resulted in 100 ASVs, which made up 9.3% of the total ASVs observed across all *Wolffia* samples (Table S4). These ASVs accounted for 22.7%, 55.0%, and 83.4% of the relative abundance in the WgPW, WgSE, and WgAS, respectively. Although the core microbiome of each category exhibited unique ASVs, four ASVs (ASV_0006, ASV_0021, ASV_0050, ASV_0095) were shared across all categories (Fig. 4a). The 100 ASVs represented 7 bacterial phyla, 33 families, and 55 genera (Fig. 4b), with *Proteobacteria* being the most abundant and consistently conserved across all categories. Among the 33 families, six families—*Beijerinckiaceae*,* Caulobacteraceae*,* Comamonadaceae*,* Methylophilaceae*,* Rhizobiaceae*, and *Sphingomonadaceae—*were consistently conserved and identified as core taxa in *Wolffia* microbiome. At the genus level, 7 of the 55 genera were consistently found in high abundance across all categories, namely *Allorhizobium-Neorhizobium-Pararhizobium-Rhizobium*,* Bosea*,* Brevundimonas*,* Caulobacter*,* Hydrogenophaga*,* Methylophilus*, and *Porphyrobacter*, with higher abundance in WgPW compared to WgSE and WgAS. Each *Wolffia* category also had specific genera that were more abundant (Fig. 4b), with WgSE and WgAS exhibiting similar high-abundance clusters.

### Potential factors influencing the shaping of bacterial communities

**Fig. 5:**
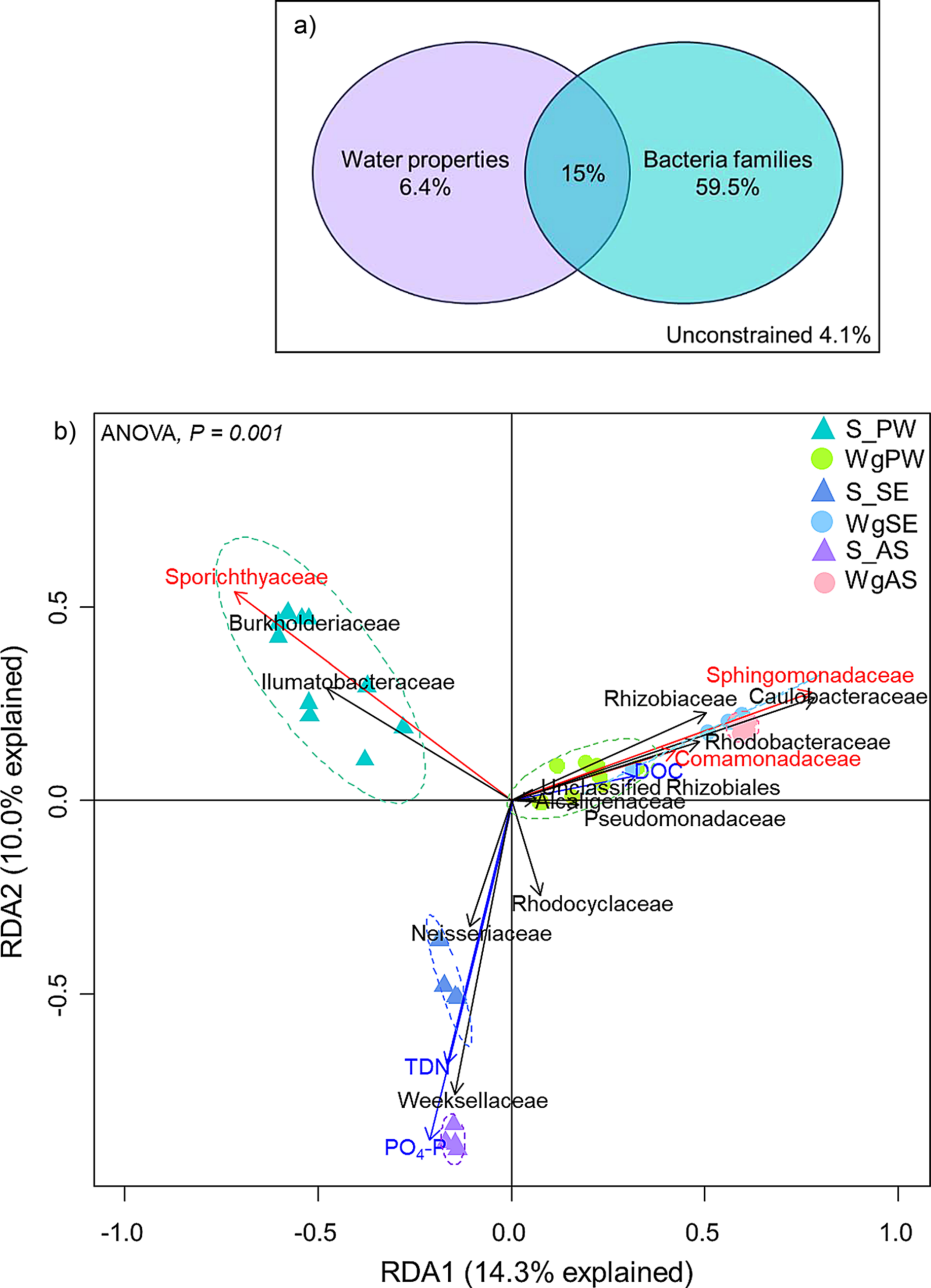
Potential factors driving community structure in microbiome. **a**) Variation partitioning analysis of bacterial diversities among the *Wolffia* and original microbial sources samples explained by water properties and bacteria families, **b**) redundancy analysis included statistically significant explanatory variables responded to the microbiome structure (ANOVA, *p* = 0.001). The significant factors were selected by forward selection indicated in red (bacteria families) and blue (environmental factors) colors. The length of each arrow indicates the contribution of parameters to the structural variation

Given the distinct structural distribution between microbial sources and *Wolffia* microbiota, an RDA was conducted to explore potential factors influencing community variation, including 3 environmental factors and 29 bacterial families (Table S5), which significantly explained 95.88% of the total variation in community structure (ANOVA, *p* < 0.001; Fig. 5a). The presence of bacterial families among the *Wolffia* communities (69.9%) strongly contributed to community dissimilarity, more than water quality. Concentrations of TDN and PO_4_–P and the presence of *Sporichthyaceae* were the three most significant factors (Fig. 5b). *Burkholderiaceae* and *Ilumatobacteraceae* positively correlated with *Sporichthyaceae*, orienting S_PW toward the top left direction. Conversely, the presence of *Neisseriaceae* and *Weeksellaceae* and two environmental factors drove the community of S_SE and S_AS toward the lower-left direction.

In contrast, the structure of *Wolffia* microbiota (WgPW, WgSE, and WgAS) congregated on the right driven by DOC, and positively correlated with the presence of eight bacterial families, with *Sphingomonadaceae* and *Comamonadaceae* being the two most important factors. These findings contributed to the distinctiveness of *Wolffia* microbiome from their microbial sources, supporting the PCoA analysis.

### Functional properties of bacterial communities

**Fig. 6:**
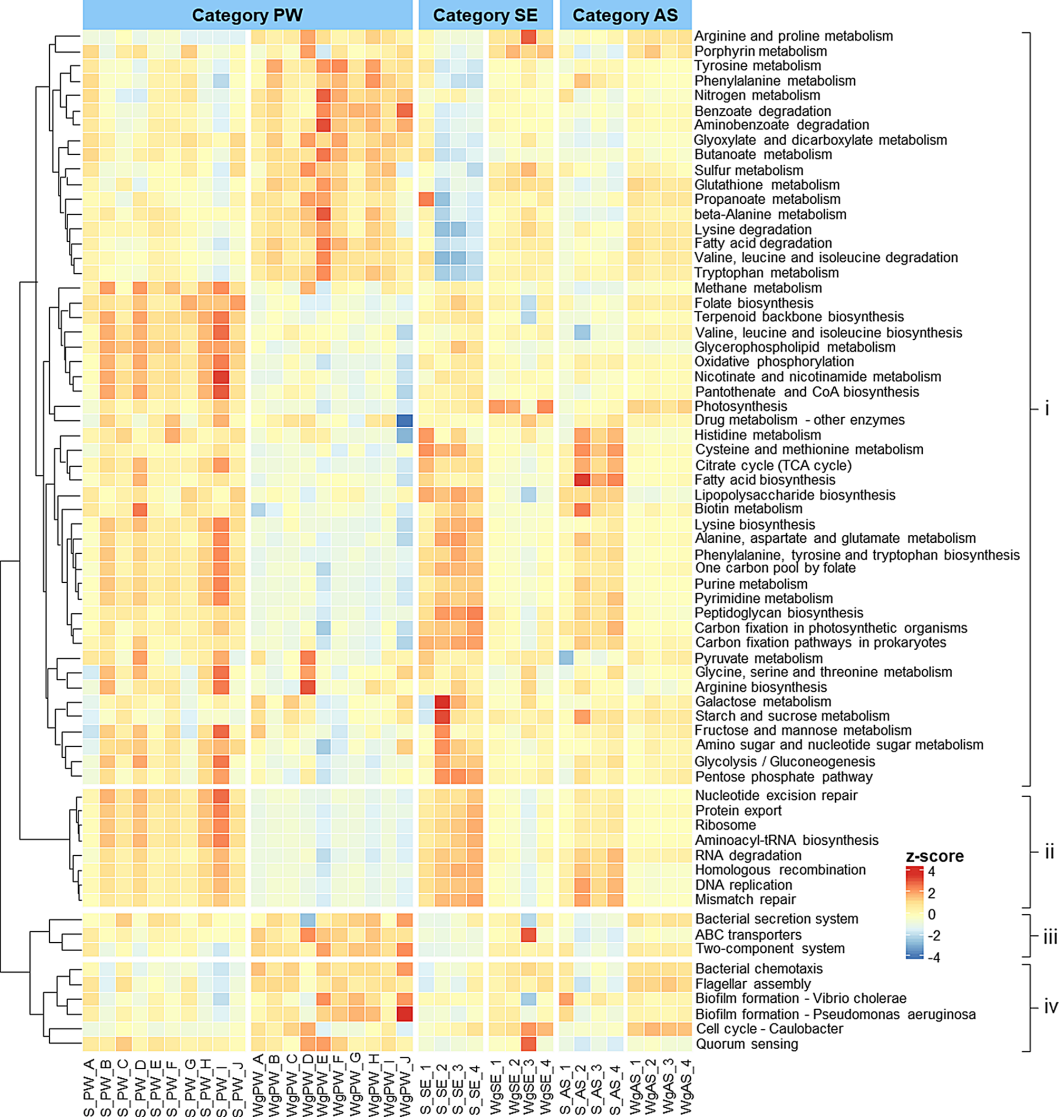
Functional properties of bacterial communities of *Wolffia* and microbial sources predicted by PICRUSt2 categorized into: (i) metabolism, (ii) genetic information processing, (iii) environmental information processing, and (iv) cellular processes. Relative abundance of gene categories was compared among the communities and the z-score is shown

The predicted functional properties of the *Wolffia* microbiome, based on the KEGG orthologs, showed notable differences from their microbial sources, with no significant variation among *Wolffia* microbiomes across the three categories. Genes involved in motility and cellular community processes, such as bacterial chemotaxis, flagellar assembly, biofilm formation, and quorum sensing, were significantly enriched in *Wolffia* microbiomes (Fig. 6). Additionally, genes associated with bacterial secretion systems, ABC transporters, and two-component systems related to membrane transport and signal transduction were enriched in *Wolffia* microbiome. Metabolic pathways linked to xenobiotic biodegradation, including amino-/benzoate degradation and glyoxylate and dicarboxylate metabolism, as well as pathways related to amino acid, energy, and carbohydrate metabolism (i.e., nitrogen/sulfur, propanoate, and butanoate metabolism), were also more abundant. Furthermore, the *Wolffia* microbiome significantly enriched genes associated with porphyrin metabolism.

### Potential key players associated with *Wolffia* growth

**Fig. 7:**
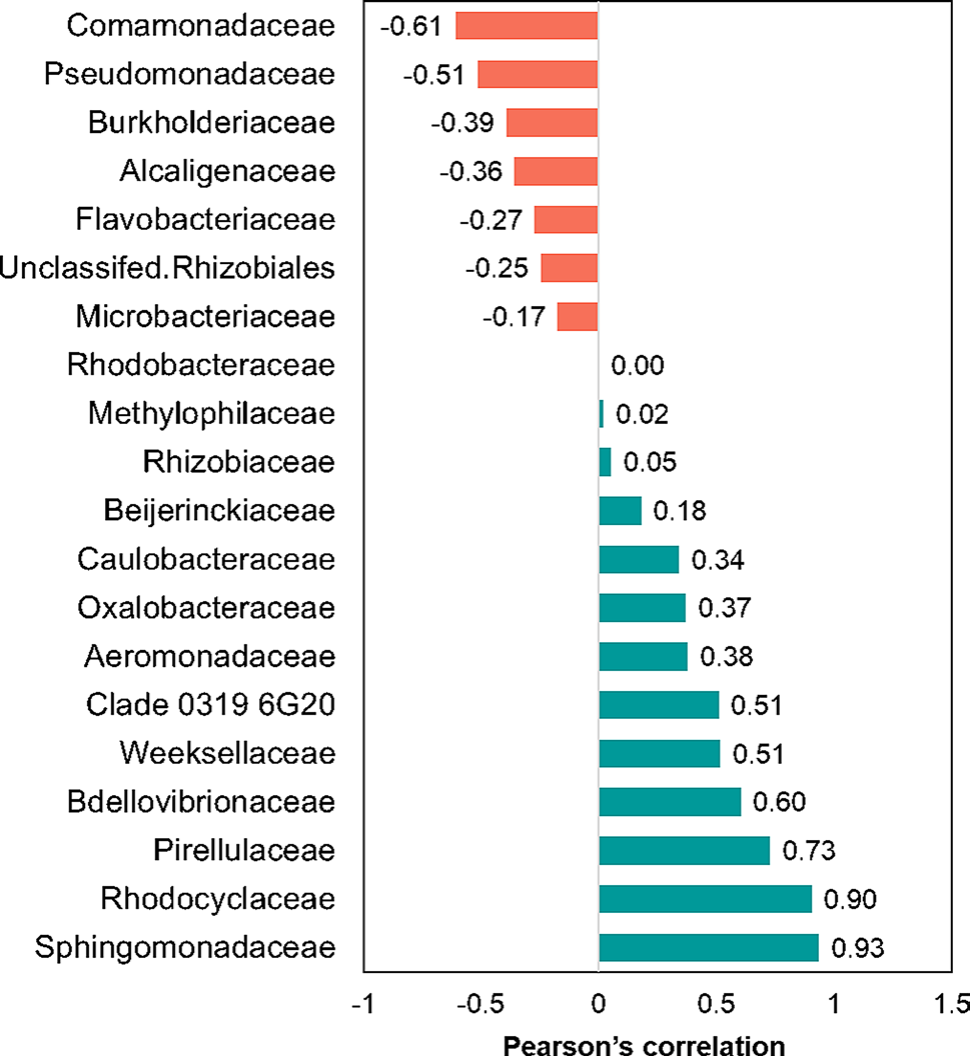
Positive/negative correlations between *Wolffia* growth and bacterial taxa considering as potential key families associated with *Wolffia* growth

A correlation analysis examining the relationship between bacterial family abundance and *Wolffia* growth was performed to better understand the key taxa influencing *Wolffia* growth (Fig. S6). The analysis found a positive correlation between *Wolffia* growth and 12 bacterial families (Fig. 7). Notably, *Sphingomonadaceae* and *Rhodocyclaceae* were strongly associated with enhanced growth. This was consistent with taxonomic distribution and EPG results, showing their enrichment in promoting communities. Interestingly, *Bdellovibrionaceae*, a family of obligate predatory bacteria, also correlated positively with *Wolffia* growth. Conversely, seven bacterial families, including *Burkholderiaceae*,* Comamonadaceae*, and *Pseudomonadaceae*, were negatively associated with *Wolffia* growth due to their dominance in inhibitory communities. Although *Comamonadaceae* was present in promoting categories (WgSE and WgAS), its abundance was lower than in WgPW, suggesting that other dominant families mitigated its effects.

## Discussion

This study demonstrated that bacterial communities derived from AS and SE potentially promote *Wolffia* growth (Fig. 1). Given that the duckweed growth is determined by the integrated activities of PGPB and PGIB in its microbiome [22, 43, 44], municipal wastewater environments may provide diverse or robust PGPB for *Wolffia*. Notably, our results suggested that PW-derived bacteria communities may not be effective for the growth of *Wolffia*. This was inconsistent with the previous findings in *Lemna minor* reporting that most of PW-derived bacterial communities exhibited promotive or neutral effects [22]. The presence of roots of duckweeds, which alter their interaction with bacterial communities, may cause different effects on *Wolffia* and *L. minor*, although further studies are required.

*Wolffia* selectively recruited and assembled bacterial communities from the inoculated microbial sources to shape its microbiome (Fig. S3), which exhibited lower diversity compared to its microbial sources (Fig. 2). This agrees with previous findings in rootless and rooted duckweeds cultured with various microbial sources [19, 30]. Among the three categories of *Wolffia* microbiome, the PW-derived microbiome had the lowest diversity, challenging our initial expectations and contradicting the findings of Inoue et al. [19]. Possibly, microbes in municipal wastewater environments may possess superior colonization abilities and a stronger response to plant exudates, allowing them to adhere more effectively to the *Wolffia* surface and occupy ecological niches [45].

*Wolffia* microbiome exhibited a distinct community composition from their microbial sources because the enrichment of specific taxa (Fig. 3, Fig. S4). *Proteobacteria* comprised most of the *Wolffia* microbiome across all microbial sources, while phyla such as *Actinobacteriota* and *Bacteroidota* were abundant in the microbial sources but diminished in *Wolffia* microbiome. The dominance of *Proteobacteria* aligns with the findings in *Wolffia australiana* and rooted duckweeds [18–20, 30, 46]. Herein, *Planctomycetota* emerged as a unique dominant phylum in *Wolffia* microbiota cultivated with AS-derived microbes. To the best of our knowledge, *Planctomycetota* has not been previously reported as a dominant phylum in duckweed-associated microbiomes.

The dominant bacterial families in the *Wolffia* microbiome exhibited distinct patterns depending on the microbial source (Fig. 3). Nevertheless, *Comamonadaceae*, *Caulobacteraceae*, and *Rhizobiaceae* were consistent dominant families. The other unique dominant families included *Burkholderiaceae* and *Pseudomonadaceae* specific to PW-derived *Wolffia* microbiota, *Rhodocyclaceae* and *Sphingomonadaceae* in municipal wastewater (SE/AS)-derived *Wolffia* microbiota, *Beijerinckiaceae* in SE-derived *Wolffia* microbiota, and *Pirellulaceae* in AS-derived *Wolffia* microbiota. Most of the dominant families identified in the *Wolffia* microbiome in this study were consistent with those found in rooted duckweeds, with the exception of *Beijerinckiaceae* [18–20]: hence, these bacterial families are commonly dominant groups in duckweeds, irrespective of the presence of roots and microbial sources. Importantly, this study reflects the apparent phenomena of microbiome recruitment in duckweeds, as *Pseudomonadaceae* and *Rhizobiaceae* were enriched in the *Wolffia* microbiome, even though they were undetectable in the corresponding microbial sources. This was most evident in the PW-derived *Wolffia* microbiota. The selection of those families is likely due to the fact that they are part of the core microbiome in duckweeds [47, 48] and are likely skilled in adaptation to the plant environment, leading to their preferential colonization and dominance in *Wolffia* microbiome, with complicated interactions in microbial networks, regardless of their abundance in surrounding environments [49].

Despite the distinct dominant families across different microbial sources, *Beijerinckiaceae*,* Caulobacteraceae*,* Comamonadaceae*,* Methylophilaceae*,* Rhizobiaceae*, and *Sphingomonadaceae* were consistently conserved and identified as core taxa in the *Wolffia* microbiome (Table. S5, Fig. 4). These core families generally resemble those commonly found in the rooted duckweed microbiome [18–20, 46]. Exceptionally, *Beijerinckiaceae* was uniquely identified as a core taxon in *Wolffia* microbiome, which are discussed below in details. These taxa belong to *Proteobacteria*, a phylum commonly associated with plant microbiota, where it exhibits symbiotic, harmful, or neutral relationships with the host [50]. Additionally, as the genera, *Allorhizobium-Neorhizobium-Pararhizobium-Rhizobium*,* Bosea*,* Brevundimonas*,* Caulobacter*,* Hydrogenophaga*,* Methylophilus*, and *Porphyrobacter* were consistently found in the *Wolffia* microbiome, suggesting their role as “stable” core taxa. The first three genera were identified as stable core taxa in natural *Wolffia* and shared with other duckweeds. Specifically, *Allorhizobium-Neorhizobium-Pararhizobium-Rhizobium* may serve as a strongly conserved core taxon, as it is consistently maintained in *Wolffia* even under nutrient-deficient conditions [20]. Meanwhile, *Bosea*,* Brevundimonas*,* Caulobacter*, and *Methylophilus* were newly reported here as stable core taxa in *Wolffia*, suggesting that it has unique core taxa and expanding our understanding of its core microbiome. The findings reflect that microbial community assemblage follows structural principles rather than occurring randomly or varying across microbial sources [51, 52]. This stability indicates that microbiome assemblage is rather a deterministic process that is independent of the initial microbial abundance in the source microbiome. The uniqueness of the *Wolffia* core microbiome is likely due to niche differences between *Wolffia* and rooted duckweed, which may contribute to selective filtering and recruitment, shaping resource availability and promoting the establishment of specific taxa. Given that niche ecology is essential in shaping microbial communities [47, 53–55], it may determine the colonization of specific microorganisms in the *Wolffia* microbiome. This phenomenon, previously observed in terrestrial plants [54], may also occur in duckweed. Xie et al. [30] demonstrated that bacteria primarily colonized reproductive pockets (where daughter and mother fronds attach) and stomata in *W. australiana*, whereas bacteria densely aggregated beneath fronds, around roots, and at the root base in rooted duckweed [45]. Furthermore, differences in exudate secretion driven by distinct morphology may cause unique plant-microbe interactions, shaping a distinct microbiome [56, 57]. These support the idea that the distinct morphology and physiology of rootless duckweed to those of rooted duckweed contribute to the formation of a unique core microbiome. However, this study defined the core microbiome based on the occurrence frequency of ASVs in each microbial source category, and the sample size in categories SE and AS was limited. Expanding the sample size is required to further validate the core microbiome in rootless duckweed.

The discrepancy in community structure between microbial sources and *Wolffia* microbiome is influenced by several factors, including fluctuations in the surrounding environment and interactions within different microbial communities [52, 58]. The RDA found that while the communities of microbial sources are diverse, the *Wolffia* microbiota are more clustered (Fig. 5). The presence of specific bacterial families such as *Sphingomonadaceae* and *Comamonadaceae* and the DOC concentration appears to be key determinants in shaping the *Wolffia* microbiome and differentiating it from microbial sources. These families were consistently enriched in *Wolffia* microbiome, indicating their significant influence on community structure. Additionally, shifts in DOC concentrations, representing the abundance of carbon sources, alter the bacterial community structure, affecting dominant taxa, diversity, and functional potential. Easily degradable DOC supports diverse bacterial populations compared to more complex or recalcitrant organic matter [59–62].

Functional profiling revealed the enrichment of genes associated with flagella assembly, biofilm formation, bacterial chemotaxis, two-component systems, and bacterial secretion systems in the *Wolffia* microbiome (Fig. 6). These genes support bacterial movement toward the duckweed surface in response to secreted exudates and facilitate bacterial attachment [63], likely being crucial in the microbiota of rooted and rootless duckweeds [19, 20, 64]. Notably, quorum sensing (QS) and ABC transporters were specifically enriched in the *Wolffia* microbiome. QS may serve as a unique functional property supporting microbiome assembly on *Wolffia*. Given that *Wolffia* lacks roots to host bacterial communities, QS likely enables bacteria to communicate and synchronize their behavior to effectively colonize *Wolffia* surfaces. The exudates secreted by rootless duckweed may act as signaling molecules, triggering bacterial QS communication and influencing plant-microbe interactions. QS signals regulate biofilm formation in a density-dependent manner, promoting bacterial aggregation and colonization on plant surfaces [65–67]. In turn, they modulate plant growth and defense mechanisms by fostering beneficial bacterial interactions and allowing the bacteria to thrive in a symbiotic relationship [68, 69]. ABC transporters contribute to nutrient uptake, enabling bacteria to acquire essential molecules, including sugars, amino acids, and specific plant metabolites required for their survival and colonization within the host plant microbiome [70]. Some ABC transporters may be involved in sensing plant signals, allowing bacteria to adapt their behavior in response to the plant environment, facilitating beneficial plant-microbe interactions. These functional properties facilitate bacterial colonization, provide a competitive advantage of dominant taxa, and enhance bacterial adaptation. Most genes related to amino acid and carbon metabolisms were also significantly enriched in *Wolffia* microbiome. This may be due to the *Wolffia* microbiome response to utilizing metabolites secreted by *Wolffia*.

Correlation analysis revealed that *Wolffia* growth is influenced by the increase of specific dominant families (Fig. 7, Fig. S6). Particularly, the increase of *Spingomonadaceae* and *Rhodocyclaceae* had a strong positive relationship with *Wolffia* growth. This is consistent with their significant enrichment in the promoting community (WgSE and WgAS). In addition to being a key player in *Wolffia* growth, *Sphingomonadaceae* is one of the core taxa and a key factor in shaping the *Wolffia* microbiome. Members of this family are commonly found in the terrestrial plant phyllosphere and contribute to plant growth promotion, protection, stress resilience, and competitiveness [71–73]. In addition to *Sphingomonadaceae* and *Rhodocyclaceae*, *Beijerinckiaceae* was not only a unique core taxon in the *Wolffia* microbiome but also influenced *Wolffia* growth and was enriched in WgSE. To the best of our knowledge, this is the first report identifying *Beijerinckiaceae* as a keystone taxon in the duckweed-associated microbiome. *Beijerinckiaceae* is uncommon in rooted duckweed-associated microbiota; however, it is commonly found in plant rhizosphere and phyllosphere and aquatic environments [74, 75]. Members of this family are recognized as versatile bacterial groups that perform methanotrophy, methylotrophy, nitrogen fixation, and phosphate mobilization, while being essential within the phyllosphere. Additionally, they can establish their populations, outcompete other microbes, and contribute to microbial community dynamics [76, 77]. Furthermore, *Beijerinckiaceae* secrete plant regulators, amino acids, and nitrogenous compounds assimilated by plants. In turn, plant exudates provide nutrients that microbes can utilize [78–80], potentially facilitating a specific interaction between *Beijerinckiaceae* and *Wolffia*.

Another interesting aspect is that *Bdellovibrionaceae* had a positive relationship with *Wolffia* growth (Fig. 7). Inoue et al. [19] also found highly enrichment of *Bdellovibrionaceae* specifically in rootless duckweed microbiome. *Bdellovibrionaceae* is known as a group of obligate predatory bacteria that actively prey on gram-negative bacteria and has not been reported as PGPB. Obligate predatory bacteria such as *Bdellovibrionaceae* do not directly affect plant growth or compete for nutrients with heterotrophic bacteria. However, in microbial ecosystems, they contribute to shaping microbiome structure through predator-prey interactions, which in turn influence plant health [81, 82]. The evidence suggests that *Bdellovibrionaceae* is crucial as the population balancer/controller in the rootless duckweed microbiome [83].

Overall, our results suggest that *Bdellovibrionaceae*,* Beijerinckiaceae*, and *Sphingomonadaceae* may serve as “hub microorganisms” and “keystone taxa” in the *Wolffia* microbiome. These families represent core taxa, act as mediators, and strongly interact with the plant and microbes, influencing microbiome structure and plant growth; this allows them to firmly integrate into the *Wolffia* holobiont, retaining their role for an extended period. These findings suggest that members of these families may function as potential PGPB for *Wolffia* in a broad sense that can enhance its productivity directly or indirectly. To confirm this hypothesis, further studies are needed to isolate strains of the potential taxa listed in this study and clarify their plant growth-promoting activities. In addition, while this study successfully investigated the effects of diverse bacterial communities and presented the *Wolffia* microbiome, the bacterial abundance in the original microbial sources was not normalized, which might have influenced the promotion/inhibition of *Wolffia* growth. While the main microbial groups and community organization likely remain consistent, how bacterial abundance influences *Wolffia* growth and associated microorganisms represents a critical question for subsequent research. Furthermore, our correlation analysis suggested that the estimated positive and negative correlations (Fig. 7) stem not only from individual bacterial families but also from interactions between multiple groups (Fig. S6). The findings emphasize the need to clarify bacterial interactions for better understanding the functions of the *Wollfia* microbiome as a system.

## Supplementary Information

Below is the link to the electronic supplementary material.

Supplementary Material 1

Supplementary Material 2

## Data Availability

The raw sequencing data is deposited with links to BioProject accession number PRJDB18855 in the DNA Data Bank of Japan BioProject Database (DDBJ).
