# Supplementary material for: Structural and functional properties of bacterial communities associated with rootless duckweed (Wolffia globosa) and their effect on the Wolffia growth
Source: Environ Microbiome. 2025 Aug 7;20:102. doi: 10.1186/s40793-025-00759-6 (PMC12329958; doi:10.1186/s40793-025-00759-6)
Supplement: Supplementary file 1 — Supplementary Material 1 [file 40793_2025_759_MOESM1_ESM.docx]

**Supplementary Information**

**Structural and functional properties of bacterial communities associated with rootless duckweed (*Wolffia globosa*) and their effect on the *Wolffia* growth**

**Text S1**

**Formula for calculating the effect on plant growth (EPG):**

$$EPG \left( \% \right)=\frac{G\left( T \right)-G\left( C \right)}{G\left( C \right)}\times100$$

where *G(T)* is the mean of RGR, calculated from the *Wolffia* area in the co-cultivation with bacterial community after 10 d of cultivation, and *G(C)* is that of the axenic *Wolffia* served as the control. Here, standard deviation (SD) for EPG was calculated as:

$$SD\left( EPG \right)=\frac{\sqrt{\left( SD\left( G\left( T \right) \right)^{2}+SD\left( G\left( C \right) \right)^{2} \right)}}{G(C)}\times100$$

where *SD(G(T))* and *SD(G(C))* are the standard deviation of *G(T)* and *G(C)*, respectively.

**Text S2**

**Redundancy analysis.** The analysis included 32 explanatory variables, comprising 3 environmental factors (DOC, TDN, and PO_4_–P), and 29 bacterial families with a relative abundance greater than 5%. The feature table was transformed using the Hellinger transformation, and the explanatory variables were standardized prior to the analysis. Variance inflation factors (Legendre and Legendre, 2012) were calculated for the variables from the RDA using the “vif.cca” function in the vegan package to detect redundant or multicollinear variables with a recommended threshold of below 10. Permutation testing of the model was performed with 999 permutations. The significance of the model and explanatory factors was determined based on the *p* values adjusted through the permutation-based approach inherent in the “anova.cca” function, with adjusting the *p* values < 0.05 considered statistically significant.


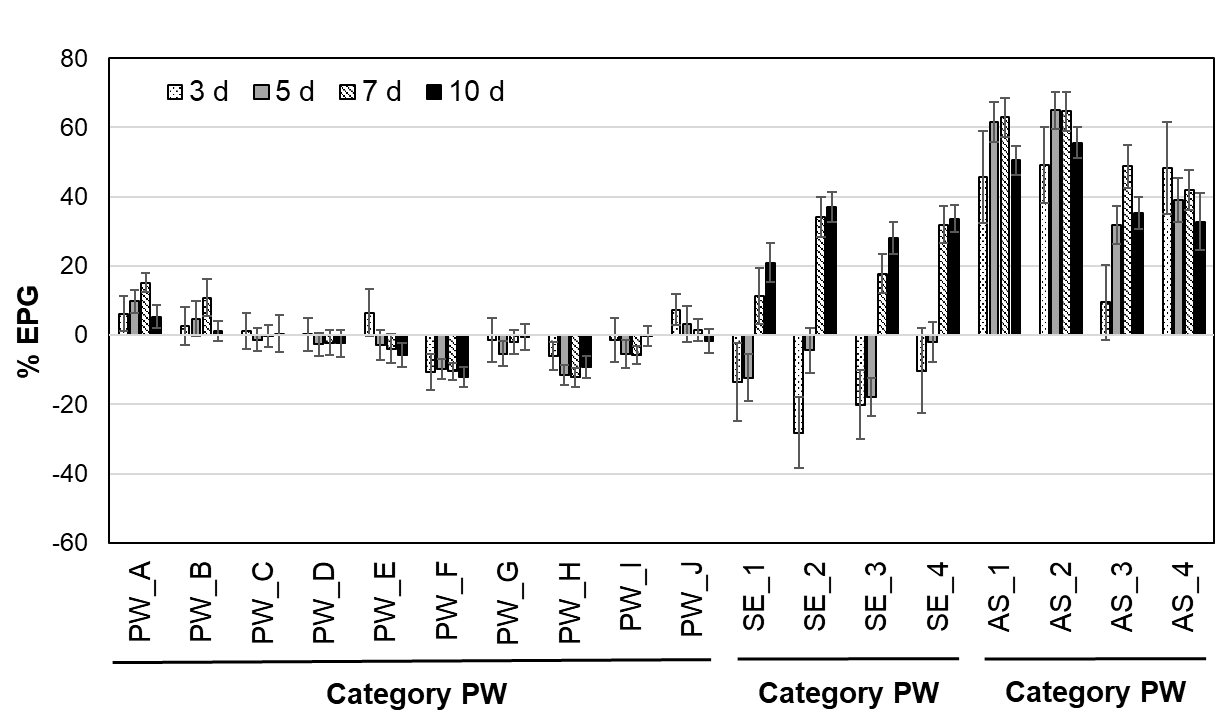


**Fig. S1** Effects on plant growth (EPG) of bacterial communities from categories PW, SE, and AS on *W. globosa* growth at 0, 3, 5, 7 and 10 d of cultivation relative to the control. Error bars represent standard deviations (*n =* 5). Bars marked with different letters indicate significant differences (*p <* 0.05, Tukey's HSD test).

**\**

**
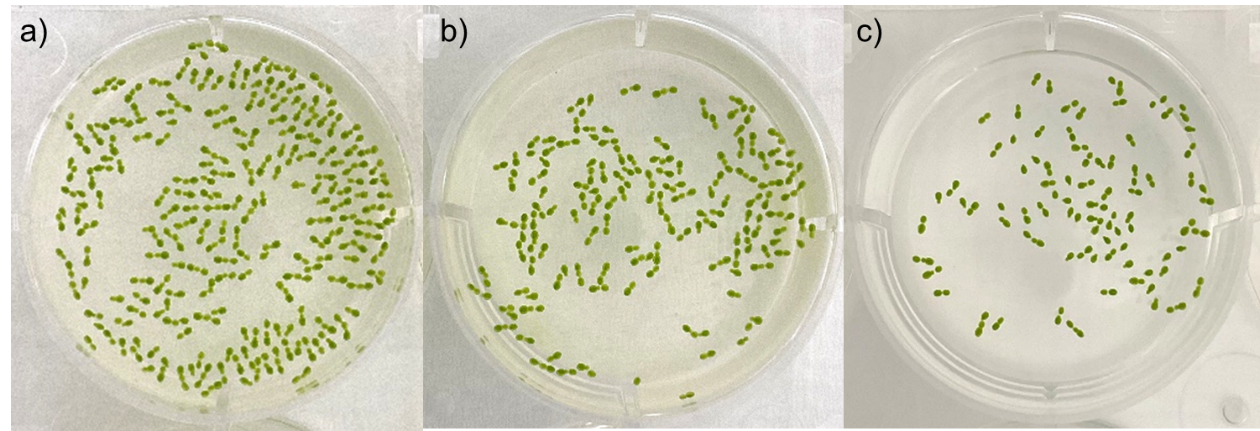
**

**Fig. S2** Representative appearance of *W. globosa* affected by bacterial communities after 10 days of cultivation: a) Promoting effect, b) Control, and c) Inhibitory effect

**Fig. S3** Venn diagram showing the percentage of recruited and unique ASVs in the *Wolffia* microbiome from its microbial sources.


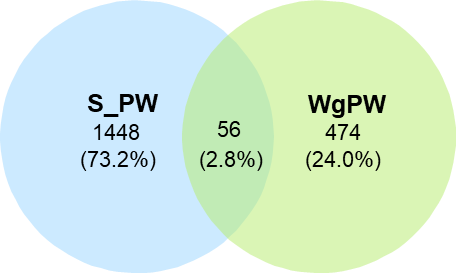

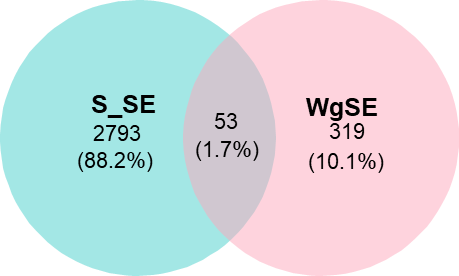

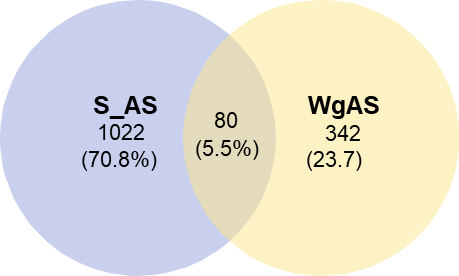


**
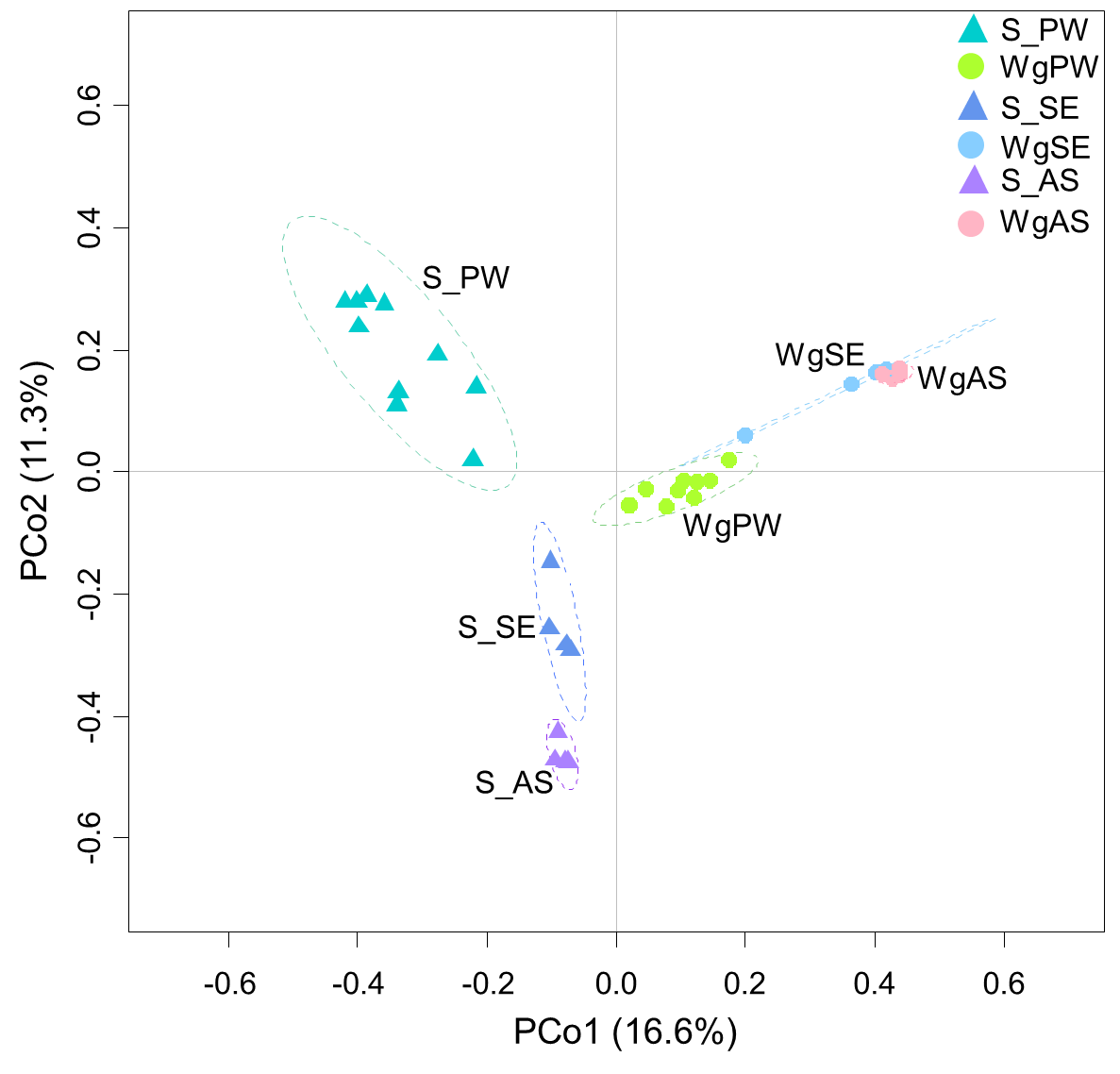
**

**Fig. S4** Principal-coordinate analysis (PCoA) based on the Bray-Curtis dissimilarity

**
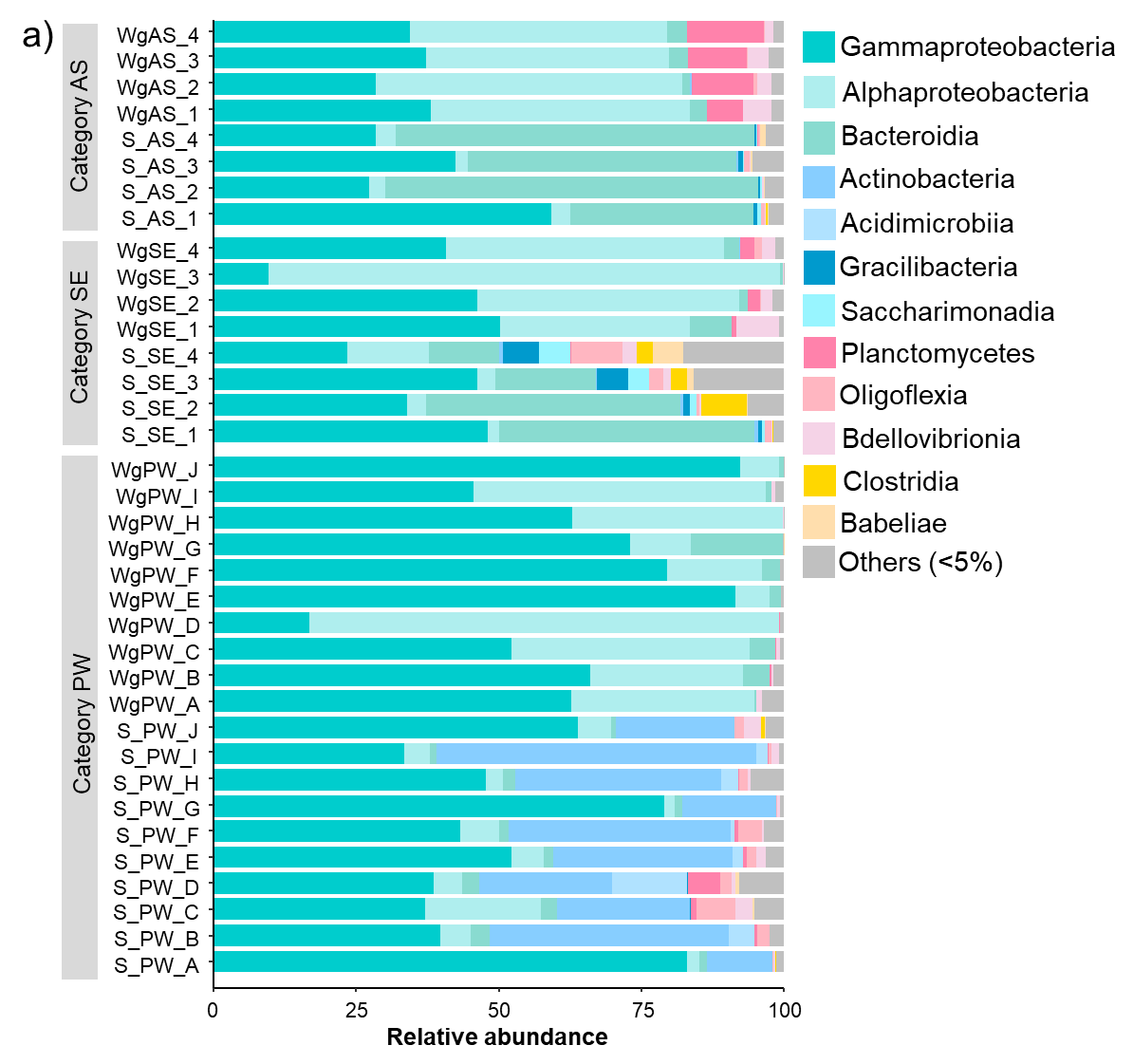

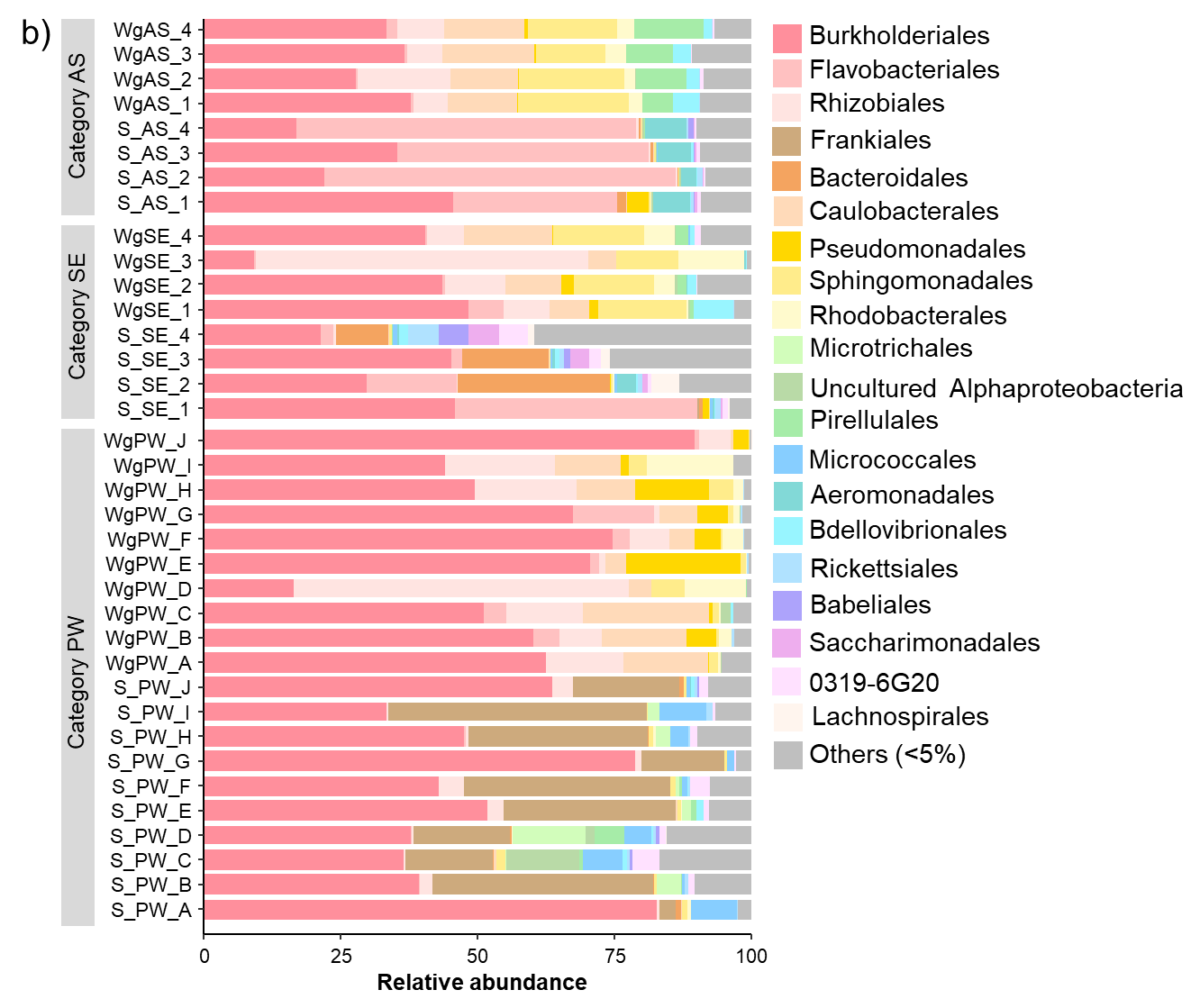
Fig. S5** Taxonomic distribution of bacterial communities in microbial sources and *Wolffia* microbiome at a) class and b) order levels. Others include all the classified and unclassified taxa with a relative abundance of less than 5% in the sample.


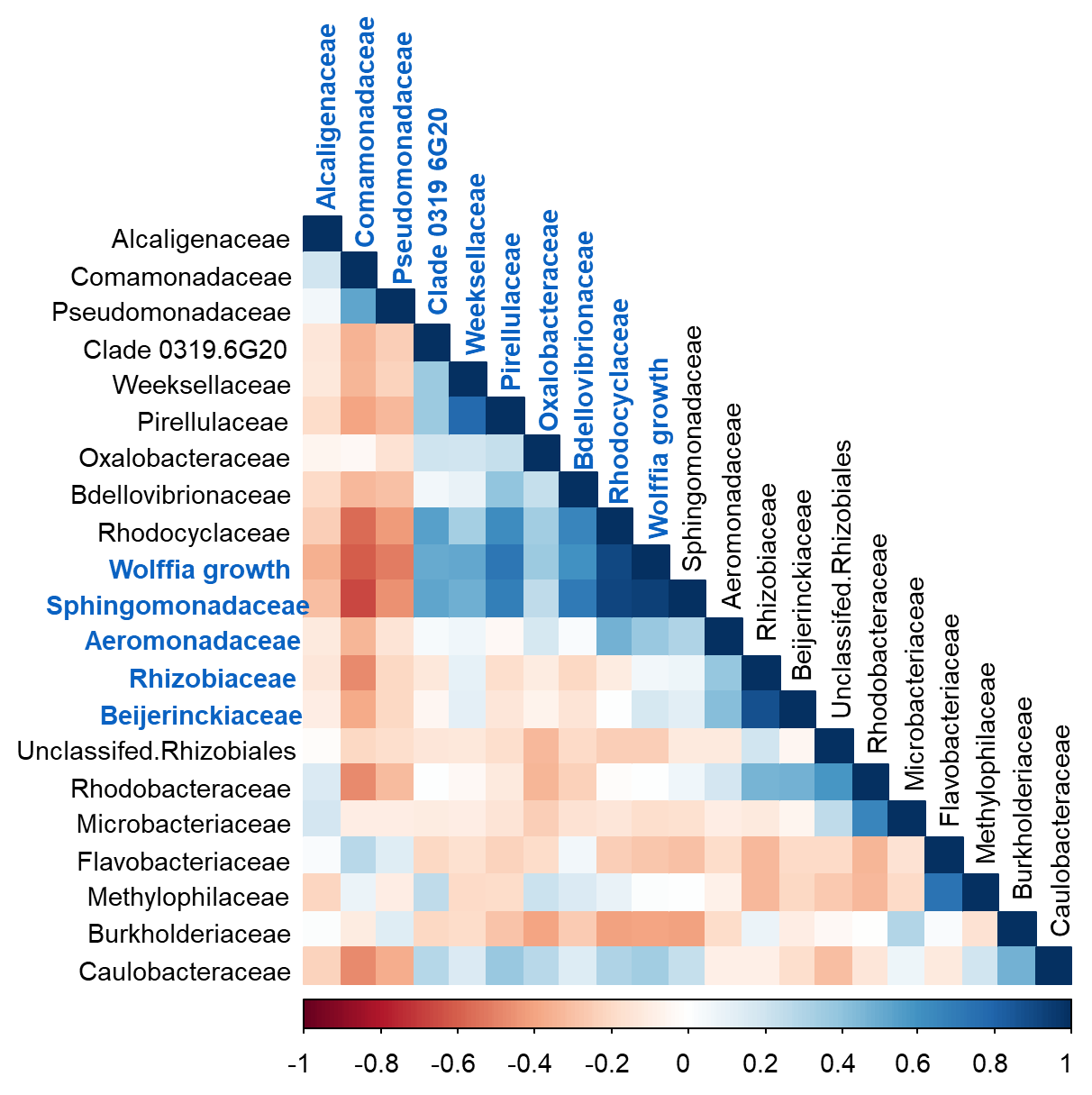
**Fig. S6** Hierarchical clustering of co-occurrence relationship between bacterial families and *Wolffia’s* growth**.** Blue and red indicate the positive and negative correlations, respectively. Families potentially associated with *Wolffia’s* growth are highlighted in bold blue text.

**References**

Legendre, P. and Legendre, L. (2012). *Numerical ecology* (Vol. 24): Elsevier.
